# Hemoadsorption contribution in neonatal cardiac surgery

**DOI:** 10.3389/fcvm.2025.1615697

**Published:** 2025-09-17

**Authors:** Isabella Molinari, Enrico Aidala, Maria Teresa Cascarano, Maria Stella Di Carlo, Cristina Rivoldini, Enrico Bonaveglio, Carlo Pace Napoleone

**Affiliations:** ^1^ Pediatric and Congenital Cardiac Surgery, Regina Margherita Children’s Hospital, Torino, Italy; ^2^ Pediatric Cardiac Anesthesiology and Intensive Care Unit, Regina Margherita Children’s Hospital, Torino, Italy

**Keywords:** pediatric cardiac surgery, neonatal cardiac surgery, hemoadsorption, cytokine, inflammatory response, cardiopulmonary bypass

## Abstract

**Background:**

Cardiopulmonary bypass (CPB) in paediatric open-heart surgery is challenging, especially in neonates and aortic arch surgery. It induces a systemic inflammatory response that can lead to significant postoperative complications, including multiorgan dysfunction, prolonged mechanical ventilation, and intensive care unit (ICU) stay. Blood purification with hemoadsorbers integrated into CPB has been proposed as a strategy to reduce these side effects. These devices adsorb cytokines from the bloodstream, trying to modulate their negative systemic effect.

**Methods:**

This retrospective study evaluates 33 neonates who underwent complex cardiac surgeries between January 2022 and January 2025 at Regina Margherita Children's Hospital. 17 of them had been treated with Jafron HA60 hemoadsorber during CPB. Biomarkers of organ damage (creatinine, lipase, aspartate transaminase, and alanine transaminase), C-reactive protein, lactates, inotropic drugs doses and a wide range of pro- and anti-inflammatory cytokines were analysed during surgery and in the intensive care unit.

**Results:**

The results showed a decrease in biomarkers of organ damage and inflammation, accompanied by a tendency toward reduction in the required dose of inotropes, ICU stays, days of mechanical ventilation, and duration of required open chest time in the treated group. A similar downward pattern was observed in cytokine levels.

**Conclusions:**

Hemoadsorption may be associated with improved clinical parameters in neonates undergoing high-risk cardiac surgery. Further large-scale studies are needed to explore these observations.

## Introduction

Open heart surgery in neonates is often accompanied by significant challenges, both in terms of surgical complexity and physiological responses induced by cardiopulmonary bypass (CPB). CPB provokes a systemic inflammatory response syndrome (SIRS), which has two phases. The first phase is secondary to surgical trauma and blood contact with nonendothelial surfaces (1–3); the second is driven by ischemia-reperfusion injury (4).

In physiological conditions, SIRS is counterbalanced by a compensatory anti-inflammatory response syndrome (CARS), which acts in parallel to limit collateral tissue injury and to promote resolution. Traditionally, this interaction was described as a sequential transition from SIRS to CARS. However, recent advances in immunology indicate that pro- and anti-inflammatory pathways are in fact activated simultaneously, forming a dynamic balance aimed at pathogen clearance and limitation of tissue damage (5, 6). This SIRS–CARS interaction is usually self-limiting and contributes to the restoration of homeostasis.

In contrast, when this regulatory equilibrium fails, the immune response becomes dysfunctional and dysregulated, leading to excessive amplification of inflammatory cascades. In this pathological context, uncontrolled release of proinflammatory mediators such as interleukin-6 (IL-6) and tumour necrosis factor-α (TNF-α) results in a cytokine storm, a state of hyperinflammation associated with endothelial injury, tissue damage, and potential multiorgan failure (7–9). Unlike the adaptive balance of SIRS and CARS, cytokine storm represents a maladaptive reaction of host defence that shifts from protection to harm.

Over the years, many solutions to this immune activation have been developed, which have led to the widespread use of corticosteroids, heparin-coated cannulas and circuits, anti-complement drugs, and, most recently, hemoadsorption therapy (3). The latter involves the use of specialised adsorbers to remove cytokines from the bloodstream and has recently emerged as a promising opportunity to reduce the adverse effects of CPB-induced inflammation in adults and in some isolated paediatric case reports (9–11).

The HA60 cartridge (Jafron Biomedial Co., Ltd., Zhuhai City, China)—the only device with a reduced priming volume specifically developed for the paediatric population—contains a porous resin made of double cross-linked styrene-divinylbenzene copolymers which can reduce inflammatory mediators and remove medium and large toxins, without compromising overall perfusion (12).

This could be of great interest in challenging surgeries that require selective cerebral perfusion and careful regulation of CPB flow (13).

This study aims to evaluate the clinical impact of the Jafron HA60 adsorber in neonates undergoing congenital heart surgery, mainly involving aortic arch, with a focus on inflammatory biomarkers, postoperative laboratory values and overall clinical outcomes.

## Materials and methods

This retrospective study evaluates 33 neonates who underwent cardiac surgeries between January 2022 and January 2025 at Regina Margherita Children's Hospital. Ethical approval for this study was obtained from the institutional ethics committee, and informed consent was obtained from the parents or legal guardians of all participants.

Seventeen patients received Jafron HA60 treatment during CPB in the specified period, depending on device availability with priority given to patients considered at higher risk of developing SIRS—such as low-weight neonates undergoing high-risk procedures, often involving aortic arch manipulation, with expected longer CPB and clamping times. The control group consisted of selected consecutive patients treated during the same period at the same institution, with comparable characteristics in terms of age, weight, diagnosis, surgical procedure complexity, and CPB and clamping times.

**Figure 1:**
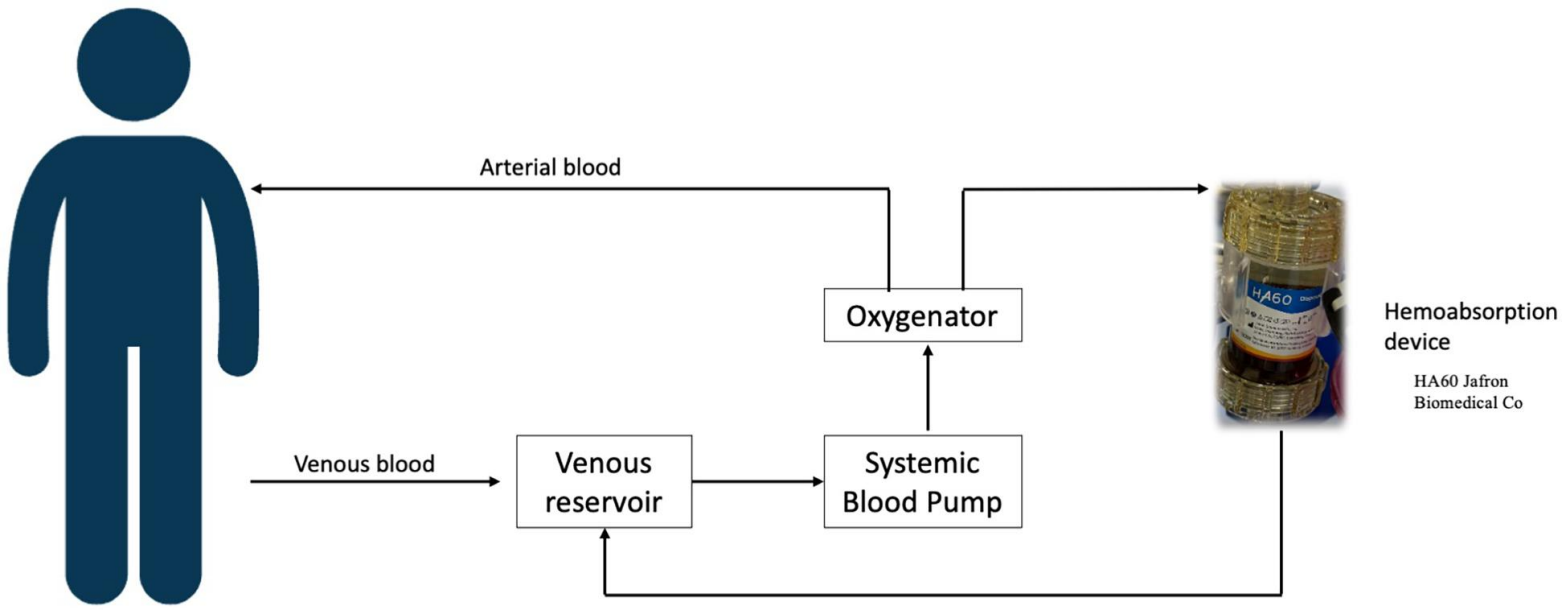
Integration of the hemoadsorption device into the cardiopulmonary bypass (CPB) circuit. Oxygenated blood is taken from the oxygenator of the heart-lung machine and goes through the adsorber to the venous reservoir.

The Jafron HA60 adsorber was connected to the extracorporeal circuit as a bypass between the oxygenator and the venous reservoir (Figure 1). Patients were connected to CPB with aortic and bicaval cannulation. Custodiol cardioplegic solution was used in all cases.

Laboratory values measuring organ damage [creatinine, lipase, aspartate transaminase (AST), and alanine transaminase (ALT)] and inflammation, such as C-reactive protein (CRP) were assessed upon arrival at the ICU and then 12, 36 and 60 h after. Lactate levels were measured immediately after disconnection from CPB, on arrival at the ICU and after 6, 12 and 24 h. The need for inotropic drugs was evaluated with the vasoactive-inotropic score (VIS) (14), which was calculated on arrival at the ICU, and then after 6, 12, 24, 48 and 72 h. A deeper analysis of cytokines (including TNF-*α* and IL-10) was obtained in serum samples from 7 of the treated patients and 2 of the control group participants at specific timepoints (Table 1).

**Table 1:** Timepoints for evaluation of cytokines. CPB: cardiopulmonary bypass, ICU: intensive care unit.

T0	Start operation
T1	Start CPB
T2	Start Selective Cerebral Perfusion (abdominal ischemia)
T3	5 min post Selective Cerebral Perfusion stop
T4	Stop CPB
T5	ICU arrival
T6	6 h post ICU arrival
T7	12 h post ICU arrival

Statistical analysis was performed using R software version 4.2.2 (https://www.r-project.org) and RStudio version 2023.6.0. Due to the limited sample size and the non-normal distribution of most of the datasets, analyses were performed exclusively using non-parametric statistical methods. Data is presented with median and interquartile range (IQR). Comparisons between independent groups were conducted using the Mann–Whitney *U*-test, while paired non-parametric comparisons were assessed with the Wilcoxon signed-rank test. Categorical variables were analysed using the Chi-square test. All statistical tests were two-tailed, and a *p*-value < 0.05 was considered statistically significant.

## Results

The two evaluated groups were comparable in terms of baseline characteristics, including median age, weight, laboratory values, and intraoperative characteristics (e.g., CPB time, aortic cross-clamp time, and need for cerebral flow) (Tables 2 and 3). The complexity of the surgical procedure was also comparable between the two groups (Table 4).

**Table 2:** Main characteristics of the jafron HA60 group and control group.

Characteristic	Control group, *N* = 16	Jafron HA60, *N* = 17	*p*-value
Baseline characteristics			
Gender			0,9
Male	8 (50%)	9 (53%)	
Female	8 (50%)	8 (47%)	
Age days			0,3
Median (IQR)	6.0 (4.8, 7.3)	7.0 (5.0, 11.0)	
Range	2.0, 10.0	3.0, 17.0	
Weight kg			0,3
Median (IQR)	2.75 (2.48, 3.10)	3.00 (2.60, 3.30)	
Range	2.10, 13.00	2.20, 3.50	
Intraoperative characteristics			
CPB duration min			0,4
Median (IQR)	176 (137, 200)	181 (150, 237)	
Range	98, 285	90, 432	
CPB flow ml/min			
Median (IQR)	515 (487, 534)	561 (479, 600)	0,3
Range	330, 1,420	444, 668	
Aortic clamp min			0,2
Median (IQR)	79 (56, 131)	111 (63, 172)	
Range	35, 207	49, 203	
Cerebral flow Yes/No			0,6
No	8 (50%)	6 (35%)	
Yes	8 (50%)	11 (65%)	
Cerebral flow ml/min			>0,9
Median (IQR)	21 (0,128)	29 (0,125)	
Range	0.350	0,180	
Clinical Outcomes			
Mechanical ventilation days			0,8
Median (IQR)	7 (6, 10)	6 (4, 15)	
Range	2, 65	3, 56	
ICU days			0,7
Median (IQR)	13 (9, 18)	11 (7, 22)	
Range	4, 80	4, 95	
Open chest Yes/No			>0.9
No	5 (31%)	5 (29%)	
Yes	11 (69%)	12 (71%)	
Open chest days			0,3
Median (IQR)	4.0 (0.0, 8.3)	2.0 (0.0, 4.0)	
Range	0.0, 15.0	0.0, 22.0	
Dead at 30 days			
No	16 (100%)	16 (94%)	>0.9
Yes	0 (0%)	1 (6%)	

**Table 3:** Clinical and laboratory results. Data are expressed as median (interquartile range, IQR).

Characteristic, median (IQR)	Control group, *N* = 16	Jafron HA60, *N* = 17	*p*-value
Creatinine			
Preoperative	0.51 (0.42, 0.73)	0.55 (0.45, 0.64)	0.9
Arrival	0.48 (0.43, 0.51)	0.38 (0.35, 0.44)	0.045
12 h	0.86 (0.78, 0.95)	0.63 (0.54, 0.65)	0.006
36 h	0.74 (0.66, 1.33)	0.83 (0.59, 1.05)	0.3
60 h	0.74 (0.63, 1.41)	0.78 (0.62, 1.03)	0.4
Lipase			
Preoperative	19 (11, 29)	11 (6, 24)	0.3
Arrival	16 (12, 24)	17 (12, 22)	>0.9
12 h	15 (11, 32)	16 (12, 27)	0.7
36 h	26 (17, 45)	25 (19, 38)	>0.9
60 h	27 (14, 36)	25 (19, 59)	0.3
AST			
Preoperative	35 (27, 40)	45 (26, 63)	0.3
Arrival	92 (59, 110)	85 (63, 96)	0.6
12 h	91 (74, 123)	94 (64, 105)	0.8
36 h	48 (40, 62)	47 (40, 57)	>0.9
60 h	32 (25, 45)	25 (23, 35)	0.4
ALT			
Preoperative	9 (7, 12)	13 (10, 20)	0.067
Arrival	9.00 (8.00, 12.00)	10.00 (9.00, 12.00)	0.093
12 h	8.0 (6.0, 10.0)	11.0 (10.0, 15.0)	0.031
36 h	5.0 (4.0, 6.5)	9.0 (7.0, 15.0)	0.016
60 h	7 (3, 13)	11 (6, 17)	0.2
LAC			
Post CPB	3.85 (2.53, 6.10)	3.40 (2.40, 4.50)	0.6
Arrival	5.40 (4.13, 7.58)	4.25 (3.00, 7.28)	0.3
6 h	4.8 (3.2, 8.4)	5.1 (2.8, 6.6)	0.8
12 h	3.00 (2.20, 4.78)	2.90 (2.30, 4.20)	0.8
24 h	2.20 (1.90, 4.13)	2.00 (1.30, 2.50)	0.2
CRP			
Preoperative	1.10 (0.35, 3.33)	2.40 (0.80, 3.60)	0.4
Arrival	2.9 (1.3, 8.6)	1.2 (1.0, 1.4)	0.02
12 h	14 (8, 24)	10 (7, 14)	0.3
36 h	16 (10, 24)	25 (16, 35)	0.2
60 h	9 (7, 17)	10 (5, 14)	0.8
VIS			
Arrival	13.0 (10.0, 15.8)	10.0 (10.0, 15.1)	0.5
6 h	14.5 (10.0, 16.3)	12.0 (10.0, 15.0)	0.5
12 h	15 (10, 18)	13 (11, 15)	0.6
24 h	14 (10, 18)	11 (8, 15)	0.1
48 h	13 (10, 19)	10 (8, 11)	0.041
72 h	13 (9, 15)	6 (5, 10)	0.023

**Table 4:** Surgical procedure performed in the jafron HA60 group of patients and in the control group.

Jafron HA60, *n* = 17(%)		Control group, *n* = 16 (%)	
Aortic arch reconstruction (+/-VSD closure, +/- supramitral membrane resection)	6 (35%)	Aortic arch reconstruction (+/-VSD closure)	6 (38%)
Norwood operation	4 (23%)	Norwood operation	5 (31%)
ASO (+/-aortic coarctation correction)	6 (35%)	ASO (+/- VSD closure)	4 (25%)
Tricuspid Valve repair	1 (6%)	Damus Stansel Kaye	1 (6%)

**Figure 2:**
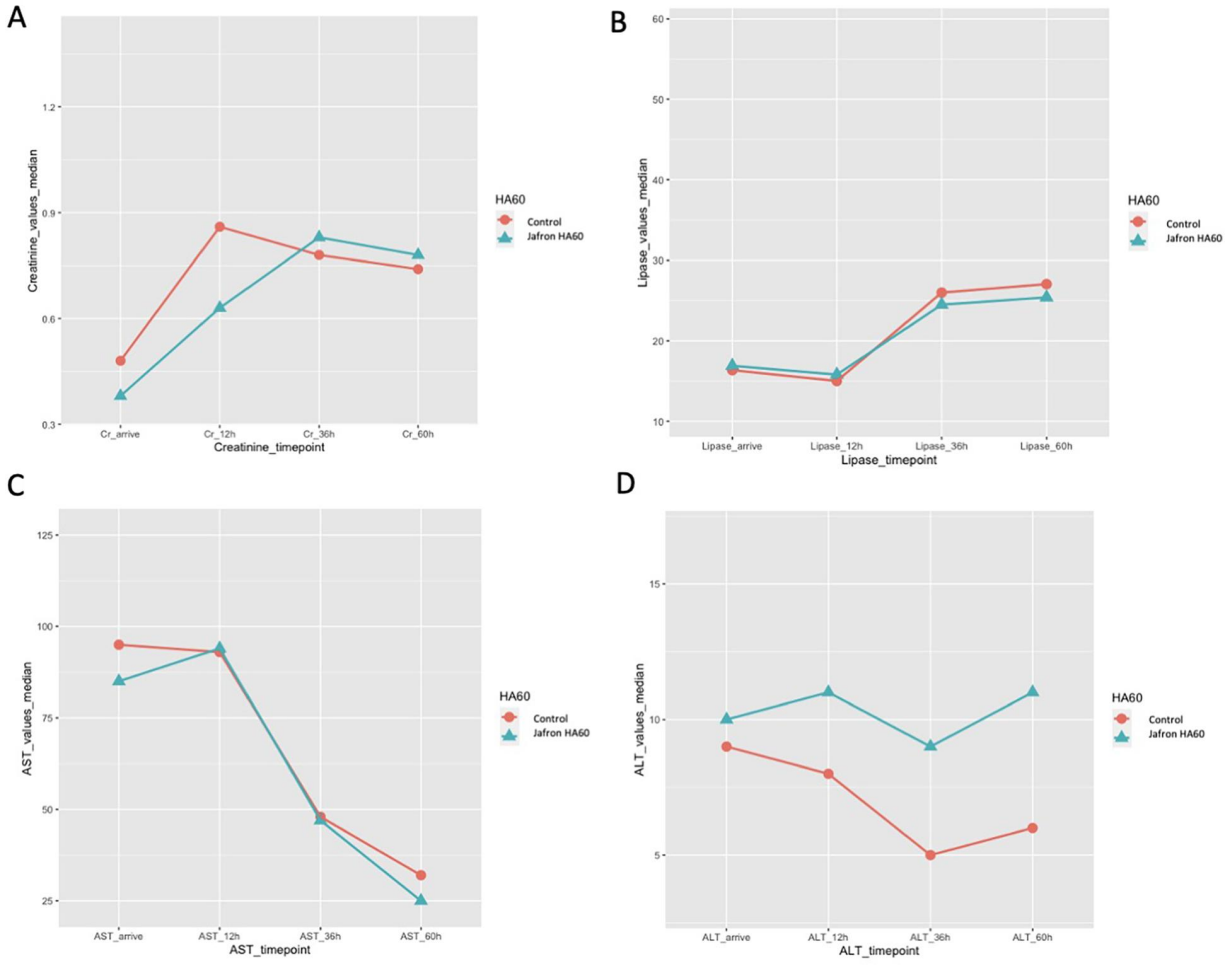
Laboratory results are shown in liner graphs. **A)** Creatinine; **B)** lipase; **C)** AST and **D)** ALT. All values were collected at admission to the ICU and at 6, 12, 36 and 60 h. For each value and statistical analysis, see Table 3.

Taking into consideration the laboratory values, the treated group presented with lower levels of creatinine, on arrival at the ICU and in the first monitored hours (creatinine on arrival 0.38 mg/dl vs. 0.48 mg/dl, *p* = 0.045; creatinine 12 h 0.63 mg/dl vs. 0.86 mg/dl, *p* = 0.006), as shown in Figure 2 and Table 3. In addition, pancreatic and hepatic damage were analysed using lipase, AST and ALT levels; however, the results were not as significant as shown for renal damage, as the treated and control group levels overlapped and sometimes reversed (Figure 2).

**Figure 3:**
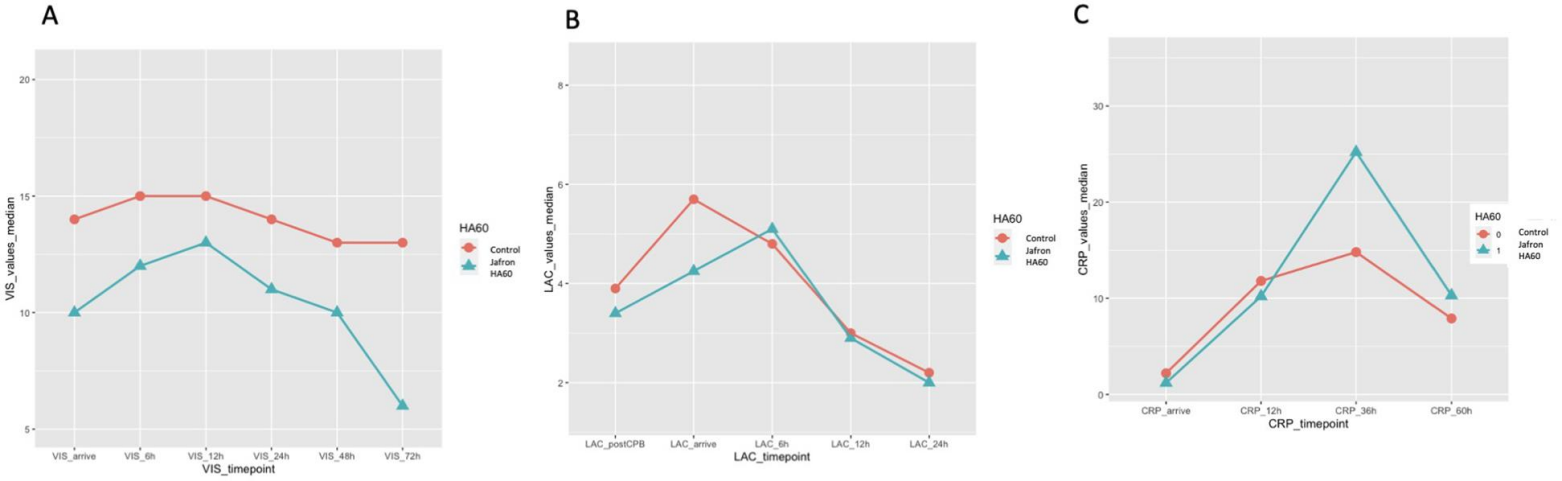
Inflammation values and VIS values are shown in linear graphs. **A)** VIS levels on arrival at ICU, at 6, 12, 24,48 and 72 h; **B)** LAC levels after CPB disconnection, on arrival at ICU, at 6, 12, 24 h; **C)** C-reactive protein (CRP) levels on arrival at ICU, at 12, 36 and 60 h. For each value and statistical analysis, see Table 3.

The inflammation monitored through C-reactive protein (CRP) revealed a significant reduction in the first hours, compared to the control group (CRP arrival 1.2 vs. 2.9 mg/dl, *p* = 0.02; CRP 12 h 10 vs. 14 mg/dl, *p* = 0.3). In addition, lactate (LAC) showed a non-significant trend toward reduction in the treated group (LAC post CPB 3.40 vs. 3.85, *p* = 0.6; LAC on arrival 4.25 vs. 5.4, *p* = 0.3) (Figure 3). VIS score was evaluated and tended to be lower in the HA60 group from the beginning of the observation period, although this difference did not reach statistical significance when compared to the control group (VIS arrive 10 vs. 13, *p* = 0.5; VIS 6 h 12 vs. 14.5, *p* = 0.5) (Figure 3).

Moreover, a tendency toward a shorter ICU stay was observed in the HA60 group compared to the control group (11 days vs. 13 days, *p* = 0.7). The need for delayed sternal closure showed a slight non-significant reduction in the HA60 group, a pattern that was also observed for mechanical ventilation time (2 vs. 4 days, *p* = 0.3; 6 vs. 7 days, *p* = 0.8).

Ultimately, the use of the hemoadsorber did not alter CPB flow or selective cerebral flow, both of which remained stable and comparable between the two groups (Table 2).

**Figure 4:**
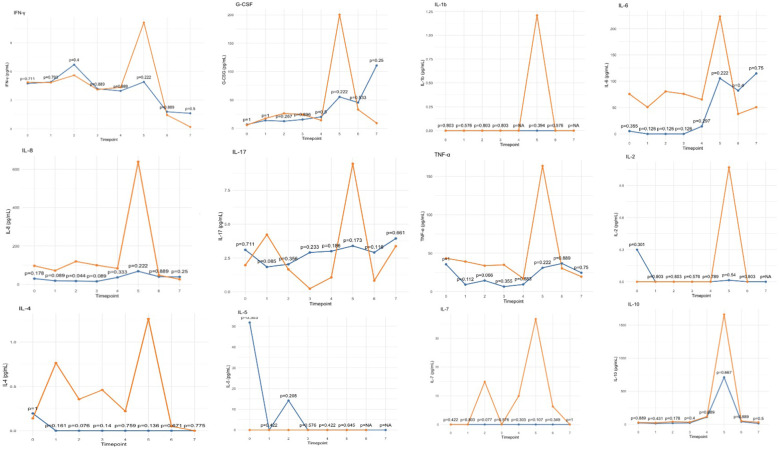
Trends in inflammatory cytokine levels, showing median values for the HA60-treated (blue) and control (orange) groups. Timepoints are represented in Table 1.

Analysis of inflammatory cytokines revealed a general tendency toward reduced levels in the treated group during CPB, followed by a modest peak upon ICU arrival, which remained lower than that observed in the control group (Figure 4). This trend was consistent across all assessed pro-and anti-inflammatory cytokines.

## Discussion

Inflammation is a critical driver of postoperative complications following CPB, particularly among neonates undergoing complex cardiac surgery. CPB triggers a systemic inflammatory response that, if unbalanced, may lead to organ dysfunction and adverse early outcomes (1–3, 8–9). Over the past decade, the understanding has evolved beyond the classical SIRS-to-CARS model: it is now recognised that both pro- and anti-inflammatory pathways are activated simultaneously, and that clinical outcomes depend on maintaining a dynamic equilibrium between these opposing responses (5, 6). Therapeutic strategies aimed at modulating, rather than suppressing, the immune response have now gained attention. In this context, hemoadsorption represents a promising approach: its advantage lies in the ability to remove both pro- and anti-inflammatory cytokines, whose excessive production can each contribute to adverse outcomes. By reducing elevated levels of these opposing mediators, hemoadsorption helps modulate a dysregulated immune response and supports the restoration of immune balance and physiological homeostasis (4, 7).

Our findings support this concept of targeted modulation rather than indiscriminate suppression. In the treated group, biomarkers of inflammation, including CRP and lactates, seemed to decrease in the first few hours, possibly indicating a faster and more effective resolution of the inflammatory response in the treated group. Therefore, the need for inotropic drugs, as indicated by the vasoactive inotropic score, tended to be lower. This tendency toward reduction was also evident in the creatinine levels, which were decreased at the first postoperative assessment, potentially suggesting an improved outcome in the treated group immediately following CPB. This trends, suggesting decreased organ damage and enhanced recovery from systemic inflammation may have contributed to shorter stays in the intensive care unit, fewer days of mechanical ventilation, and reduced need for delayed sternal closure.

The effect of this hemoadsorber, as described in literature, results from the adsorption of inflammatory mediators and toxins (12). While the comparative analysis did not show statistically significant differences, all evaluated cytokines exhibited a consistent trend toward lower levels in treated patients, particularly at ICU admission when peak concentrations were observed (Figure 4). Given the small sample size, these findings should be further explored in larger cohorts, as the limited number of patients may explain the lack of statistical significance.

All these results are in line with what has been shown so far in the literature (15, 16), with a growing number of reports describing the effective use of other hemoadsorbers in different clinical settings often related to inflammatory processes (17).

Moreover, Jafron HA60 is easy to integrate into the CPB circuit and can also be connected during CPB without interrupting bypass if serious intraoperative complications occur.

One of the key limitations of this study is the small sample size, which reduces the statistical power of the analysis and often results in a lack of statistically significant findings. In addition, the limited generalisability of the results due to the specific characteristics of the study population means that the findings may not be replicable in different populations. For these reasons, the analysis primarily focused on the observed trends rather than the absolute values. Larger randomised controlled trials are required to include a more representative sample and to definitively establish the role of hemoadsorption in cardiac surgery, especially within the paediatric population.

## Conclusion

Hemoadsorption with HA60 has shown the potential to modulate, rather than completely suppress, the CPB-induced systemic inflammatory response in neonates undergoing high-risk congenital heart surgery.

Treated patients tended to exhibit lower, although not yet statistically significant, levels of inflammatory biomarkers and cytokines, improved postoperative recovery, and shorter ICU stays. While these results are promising, larger and more comprehensive studies are needed to confirm the clinical benefits of hemoadsorption in paediatric cardiac surgery.

## Data Availability

The raw data supporting the conclusions of this article will be made available by the authors, without undue reservation.
